# Energy, Nutrient and Food Intakes of Male Shift Workers Vary According to the Schedule Type but Not the Number of Nights Worked

**DOI:** 10.3390/nu12040919

**Published:** 2020-03-27

**Authors:** Sophie Bucher Della Torre, Pascal Wild, Victor Dorribo, Brigitta Danuser, Francesca Amati

**Affiliations:** 1 Department of Nutrition and Dietetics, Geneva School of Health Sciences, HES-SO University of Applied Sciences and Arts Western Switzerland, Rue des Caroubiers 25, 1227 Carouge, Switzerland; 2 Department of Health, Work and Environment, Center for Primary Care and Public Health (Unisanté), University of Lausanne, 1011 Lausanne, Switzerland; 3 Scientific Management, INRS, 54519 Vandoeuvre-lès-Nancy, France; 4 Department of Physiology and Institute of Sport Sciences, University of Lausanne, 1005 Lausanne, Switzerland

**Keywords:** food intake, eating behavior, shift work, meal timing, meal structure

## Abstract

Shift work is associated with increased risk of chronic diseases due to circadian rhythm disruptions and behavioral changes such as in eating habits. Impact of type of shifts and number of night shifts on energy, nutrient and food intake is as yet unknown. Our goal was to analyze shift workers’ dietary intake, eating behavior and eating structure, with respect to frequency of nights worked in a given week and seven schedule types. Eating habits and dietary intakes of 65 male shift workers were analyzed in three steps based on 365 24-h food records: (1) according to the number of nights, (2) in a pooled analysis according to schedule type, and (3) in search of an interaction of the schedule and the timing of intake. Mean nutrient and food group intake during the study period did not depend on the number of nights worked. Amount and distribution of energy intake as well as quality of food, in terms of nutrient and food groups, differed depending on the type of schedule, split night shifts and recovery day (day after night shift) being the most impacted. Shift workers’ qualitative and quantitative dietary intakes varied between different schedules, indicating the need for tailored preventive interventions.

## 1. Introduction

The number of shift workers is increasing worldwide. In Europe, about one worker out of five works night shifts [1]. In Switzerland, it is estimated that more than 200,000 people regularly work at night [2]. Most studies on shift work classify shift workers as anyone working outside regular daytime hours (i.e., between approximately 7 a.m. and 6 p.m., Monday through Friday), including all people working evening, night, rotating, irregular or on-call schedules during the week and on weekends [3]. In addition to its impact on family and social life, shift work impacts health and has been associated with an increased risk of developing obesity [4,5,6,7], metabolic abnormalities [8,9] and several chronic diseases [10,11,12,13]. In France, an evidence-based report concluded that shift work impacts drowsiness, hampers sleep quality and total sleep time, and leads to metabolic syndrome. Negative effects on psychological health, cognitive performance, obesity, weight gain, type 2 diabetes, coronary heart diseases and cancer were considered probable and effects on dyslipidemias, hypertension and ischemic stroke possible [14].

Despite the observed increased risk of developing chronic diseases, mechanisms involved in these associations remain unclear. Inversion of wakefulness and sleep periods is suspected to increase the risk of disease directly through an internal disruption of circadian rhythms [15]. Behavioral changes, such as eating habits and food intake, are also frequently observed among shift workers and may act as additional risk factors [16]. An interaction between these factors has also been hypothesized [17].

Circadian rhythms are “fundamental biologic processes, nearly ubiquitous in living systems, that enable organisms to anticipate and prepare for predicable changes to the environment that occur as the earth rotates on its axis every 24 h” [18]. Circadian rhythmicity is generated by a central clock located in the suprachiasmatic nuclei (SCN) of the hypothalamus and by “peripheral clocks” located in all major tissues in the body, including the liver and pancreas [19,20]. Digestion, nutrient metabolism and energy homeostasis are regulated by the circadian activity of hormones (e.g., insulin, glucagon, corticosterone, leptin, ghrelin) and enzymes involved in cholesterol, amino acid, lipid and glucose metabolism [21,22]. From a nutritional point of view, humans are programmed to alternate between periods of food intake (day) and fasting (night) [23,24] and shifting from this, especially eating in typical fasting time, can alter health outcomes [25].

Misalignment between internal circadian rhythms and behavioral cycles can cause physiological changes and may explain the adverse health effects of shift work [19]. Shift work is also frequently associated with sleep disturbances, and shift workers accumulate sleep debt over time [26]. Lack of sleep has also been associated with an increased risk of weight gain [27] through several mechanisms [28]: (1) a decreased circulating leptin level, (2) an increased ghrelin level, and (3) an increased energy intake. Sleep debt is also associated with insulin resistance and decreased glucose tolerance [29].

To understand the observed weight gain in shift workers, researchers have focused on total energy intake, nutrients, food groups and eating behaviors [4]. A thorough systematic review including 33 observational studies revealed no association between energy, macronutrient or food group intakes and shift work [30]. However, studies included in this review revealed that shift workers differed from those working only in the daytime in timing of meals and eating patterns, indicating that shift work may influence more the timing of intake rather than the overall nutritional intake. For specific variables, the review found mixed results, with some studies pointing out that shift workers tended to eat more foods rich in saturated fatty acids (in three of five studies) and sugar-sweetened beverages (in five of eight studies). Previously, Lowden et al. (2010) reported that night workers tend to less closely follow the national nutritional recommendations [31]. For example, they eat less fruit and dietary fiber, and consume more fat, simple sugars and alcohol [31]. Shift work appears to affect patterns of eating and energy distribution throughout the day, especially with more calories consumed during the evening and night [32]. This disruption is not without consequence, as consuming a larger proportion of energy later in the day and at night has been associated with an increased risk of obesity [33]. Night workers also eat more irregularly during their shifts, with frequent small amounts (nibbling) replacing structured meals [31,32,34]. Internal sensations, such as hunger or satiety, seem to be reduced at night, but thirst does not show a circadian effect, at least in the short term [35]. On the other side of the energy balance, McHill et al. showed a reduction in total energy expenditure during night shifts, compared to day shifts [36]. Moreover, the thermic effect of food appears higher in the morning relative to late afternoon and evening [37,38]. Energy intake and expenditure during a night shift may have a carry-over effect on daytime while off-shift, complicating the calculation of mean dietary intake, by diluting any potential signal by incorporating different shift schedules [39].

Most studies investigating shift workers’ eating habits have included inter-individual comparisons of total energy intake, macronutrient intake and meal frequency and comparison of results with the intake of day workers [31,40]. Fewer studies employed within-subject comparisons, collected data regarding consecutive shifts and free days [31] or reported analyses at the level of food items, macronutrient proportion, micronutrient intake or eating behavior [41]. Importantly, shift workers are a very heterogeneous population with a variety of schedules and work organizations. Whether eating habits vary according to the number of nights worked per week and schedule type is not explored and may explain inconclusive or discrepant results previously reported. In addition, an analysis of day-to-day food intake should allow us to better identify targets of preventive interventions.

Therefore, the aim of the present study is to quantitatively and qualitatively analyze and differentiate shift workers’ energy, macro- and micronutrient and food group intakes, eating behavior and eating structure, with respect to the number of nights and types of schedules.

## 2. Methods

### 2.1. Study Design, Sample and Procedures

We analyzed energy, food and nutrient intakes as well as eating behavior of shift workers taking into account their work schedule. For the first step, we compared mean dietary intakes of the study period (5 or 6 consecutive days) with respect to the number of nights worked during that period. In the second step, we pooled all participants’ 24-h dietary intakes and analyzed their association with the type of schedule. Finally, we evaluated the effect of schedule type on the timing of energy intake.

Sixty-nine shift workers enrolled in the study, but four of them did not finish it. The final sample included 65 male participants from three workplaces. All were shift workers but had a variety of work schedule types. Twenty-eight were airport firefighters with regular 12-h shifts (7 a.m.–7 p.m. and 7 p.m.–7 a.m.). They worked two “days”, then two “nights”, followed by one day of recovery and three days off. Twenty-nine were police officers, of whom most followed a pattern of two “days” (6 a.m.–5 p.m.), one “split night” (6 a.m.–12 p.m. and 7 p.m.–6 a.m.), one day of recovery and two days off. The organization of their work was very irregular and dependent on their activities. Eight were employees of an international airport with very irregular work and meal schedules. Their earliest schedule could start at 4 a.m. and the latest one finish at 1 a.m. In the three workplaces (airport, airport fire department, regional police department), located in the region of Geneva in Switzerland, all shift workers received information about the study and were invited to participate. Inclusion criteria were schedules including work between 8 p.m. and 6 a.m., age between 18 and 65, and employment for at least 6 months in the company with this type of work schedule. Measurements took place during work hours. All participants signed an informed consent letter and the Geneva Cantonal Ethics Committee on Research Involving Humans approved this research project (2016-00077).

### 2.2. Food, Nutrient and Energy Intake

Participants used a previously validated electronic food record [42] to log food and beverage intake for 5 or 6 consecutive days, beginning on the first day of their shift. They received an oral and written instruction on the food recording and were asked to follow their habitual diet. For each food or drink consumed, participants indicated the date, time of day, and type of food or beverage. To report portion sizes, they used the propositions within the tool (e.g., for bread “a slice (35 g)” or for banana “1 piece (160 g)”) or entered the exact weight of the consumed food. An experienced dietitian reviewed the records with participants a few days after the end of the data collection. Nutrients and energy intakes were calculated using data from the Swiss Food Database Composition [43]. Food group servings were calculated according to the Swiss Food Guide Pyramid [44]. Total energy, nutrients and food group intakes were computed day by day (5 a.m.–5 a.m.) and by 8-h periods (5 a.m. to 1 p.m., 1 p.m. to 9 p.m. and 9 p.m. to 5 a.m.). The number of “eating occasions” was calculated, representing the number of times a participant ate or drunk anything else than plain or sparkling water. To assess eating habits globally, the PNNS-GS (Programme National Nutrition Santé Guideline Score) score [45] was used to assess compliance with food intake recommendations. This validated score is based on the French recommendations, which are similar to the Swiss ones. One participant did not compete the food record.

### 2.3. Eating Behavior

We assessed eating behavior using two instruments: The French-validated Dutch Eating Behavior Questionnaire (DEBQ) [46] and the Intuitive Eating Scale (IES-2) [47]. The DEBQ assesses three eating styles: (1) emotional eating, which is eating in response to negative emotions as an atypical response to distress; (2) external eating, which is eating in response to food-related stimuli regardless of the internal status of hunger and satiety and (3) restrained eating. High dietary restraint has been associated with increased impulsivity and disinhibited eating [48]. 

The French version of the IES-2 assesses three central features of intuitive eating, which have been defined as “eating in response to physiological hunger and satiety cues rather than external and/or emotional cues, in addition to low preoccupation with food” [47]: (1) eating for physical rather than emotional reasons, (2) unconditional permission to eat and (3) reliance on hunger and satiety cues. A higher score indicates greater levels of intuitive eating or its dimensions. Six participants did not fill the questionnaires.

### 2.4. Anthropometrics

Participants were weighed with clothes (empty pockets) but without shoes on the same calibrated scale (SECA 877), and their weight was rounded to the nearest 0.1 kg. We took 1 kg off the measured weight to account for clothes. Height was rounded to the nearest 0.1 cm, using a portable stadiometer (SECA 217). Body mass index (BMI) was calculated and we used the cut-offs of the World Health Organization to define underweight (BMI < 18.5 kg/m^2^), normal weight (BMI 18.5–24–9), overweight (25.0–29.9) and obesity (>30.0) [49]. Body composition was assessed using a 4-point bioelectrical impedance analysis and the local validated Geneva formula [50]. Two trained investigators performed all the anthropometric measures.

### 2.5. Statistical Analysis

Participants were separated into three groups based on the number of nights worked during the study period: one night, two nights, and three or more nights. We described mean nutrient and food group intakes during the study period (5 to 6 days), eating behavior scores and anthropometrics (mean ± SD) and compared results between the three groups adjusting for the workplace to take into account a possible confounding effect by the workplace, using a multiple (two-way) linear model.

Then, in a pooled analysis based on 365 24-h food records, nutrients and food group intakes per 24-h period (5 a.m.–5 a.m.) were compared according to the different schedule types. For this, the 56 schedule types were grouped in 7 categories: day, evening, half-night (ending between 0–1 a.m.), split-night (6 a.m.–12 p.m. and 7 p.m.–6 a.m.), night, day following night (recovery from night) and day off. These comparisons were done using a linear mixed model with the subject ID as a random effect for each outcome. We performed a Scheffe post hoc analysis to explore differences between multiple group means while controlling the experiment-wise error rate.

A third analysis described the differential effect of the schedule type according to the 8-h period. As this analysis involved repeated measures per subject, it relied on linear mixed models with the subject ID as a random effect. The independent variables were the schedule type in interaction with 8-h periods (5 a.m.–1 p.m., 1 p.m.–9 p.m., 9 p.m.–5 a.m.). These particular 8-h periods were chosen within the 5 a.m.–5 a.m. 24-h period and to allow comparison across equal blocks covering the morning, the afternoon and evening, and the night. The results are reported as the model-predicted margins means for each combination of schedule type and 8-h period.

Lastly, as the PNNS-GS score does not apply to single day intake, we created a daily qualitative healthy eating factorial score based on absolute intake of added sugars and alcohol as well as the following variables normalized for total energy intake: servings of fruits and vegetables, whole grains, meat, fish and eggs as well as saturated fatty acid and sodium intake. This daily score was compared for each schedule type using linear mixed models with the subject ID as a random effect. The results are reported as the model-predicted margin means for each combination of schedule type and 8-h period.

Descriptive results were presented as means and standard deviations for symmetrically distributed variables. For all comparisons, a *p*-value < 0.05 was considered statistically significant.

## 3. Results

Sixty-five shift workers from three workplaces, with a mean age of 38.6 (± 7.0), participated in this study. Table 1 presents participants’ characteristics and number of nights worked.

Using BMI, 38% were within the norms, 49% were overweight and 13% were obese. Using percentage of body fat mass, 52% exceeded the cut-off of 25% recommended by the American Council on Exercise [51]. Regarding their health, one participant declared having diabetes, four declared having food allergies or intolerances, eleven followed a diet (one with a dietitian, one Weight Watcher, one Diet Zone, eight not defined). Nineteen declared taking food supplements (spirulina, omega-3, multivitamins, vitamin D).

### 3.1. Analysis of Energy, Nutrient, Food Group Intakes and Eating Behavior Questionnaire between Groups

Shift workers working one, two, or three or more nights during the study period showed almost no statistically significant differences in nutrient and food group intake over the entire assessed period (Table 2).

### 3.2. Analysis of Energy, Nutrient and Food Group Intake Between Schedule Types

Table 3 details the participants’ day-by-day energy, nutrient and food group intakes for each schedule type. This pooled analysis of 365 24-h food records (5 a.m.–5 a.m.) showed large variations in total energy intake depending on schedule type, some of which were statistically significant. Participants working split nights (6 a.m.–12 p.m. and 7 p.m.–6 a.m.) had higher energy intake during their work schedule type compared to all other schedule types. In addition, participants consumed less energy on the days following nights (recovery days). When we grouped together the types of night schedules (evening, half-night, split-night and night), no significant differences emerged in energy, nutrient or food group intakes between day and night, with the exception of alcohol consumption, which was lower at night (*p* = 0.003).

**Figure 1:**
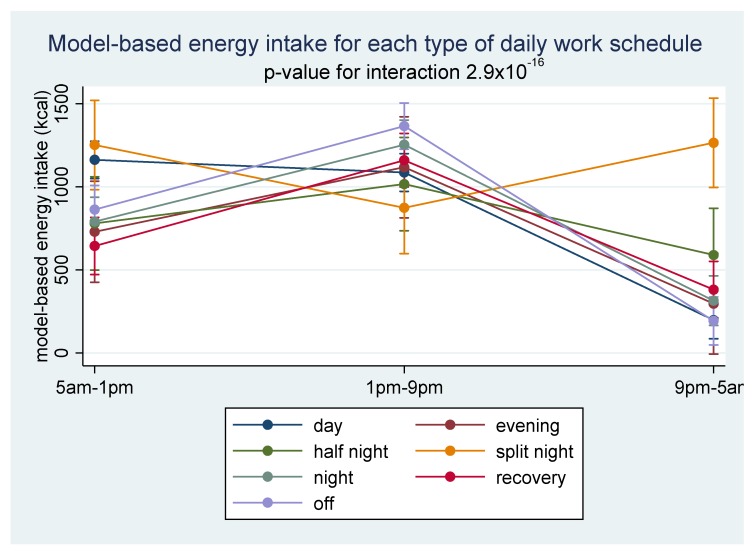
Model-predicted mean energy intake (kcal) with 95% confidence intervals, by 8-h periods according to each schedule type.

Distribution of energy intake also depended strongly on schedule type. As Figure 1 shows, during split nights, increased energy consumption in the morning (5 a.m.–1 p.m.) and at night (9 p.m.–5 a.m.) explained the higher total energy intake. The proportion of energy intake for each 8-hour period during different schedule types is presented in Supplementary Materials (Table S1).

A qualitative analysis of food intake (Table 3) revealed that during split nights, participants ate significantly more starchy foods and grains, meat, fish and eggs and dairy. They had higher intakes of fiber, sodium, calcium and magnesium. However, these differences disappeared when we normalized intakes for energy intake. On recovery days (day following nights), participants had a higher intake alcohol, compared to day shifts or night shifts.

**Figure 2:**
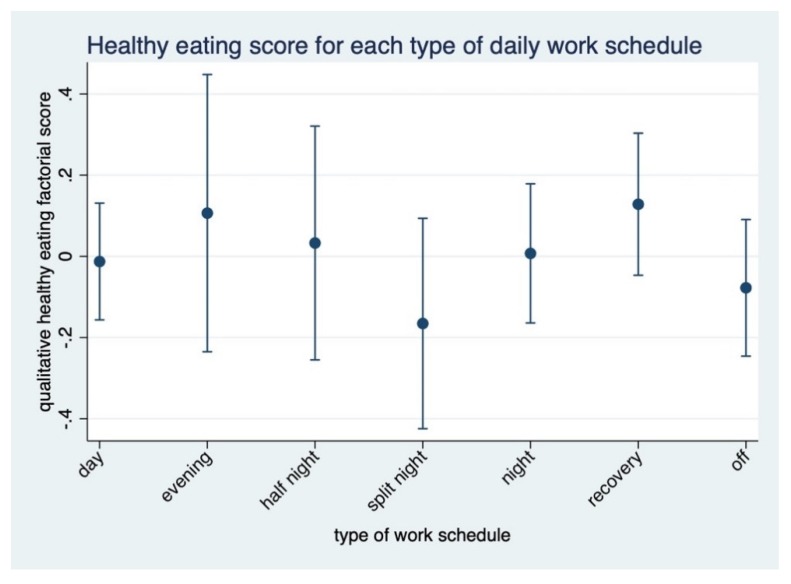
Daily qualitative healthy eating factorial score normalizing for total energy intake (positive score meaning healthier) for each schedule type.

A daily qualitative healthy eating factorial score that normalizes for total energy intake and takes into account the number of nights (Figure 2) showed that participants’ intakes on recovery days were healthier compared to other schedules (*p* < 0.05). In this analysis, the least healthy pattern was found in split nights (*p* < 0.05).

## 4. Discussion

This study aimed at analyzing shift workers’ dietary intakes, eating behavior and eating structure with respect to the number of nights worked and schedule type. In our sample, the mean dietary intakes during the study period did not depend on the number of nights. Total energy intake, distribution of energy intake through the 24-h period and quality of food intake at the nutrient and food group levels differed by schedule type. Participants working split nights consumed significantly more energy compared to all other schedule types. Days following night shifts were characterized by a low absolute intake of key nutrients and food groups compared to split nights but a higher healthy eating score when normalized for total energy intake. These results highlight several methodological issues that may explain the lack of convincing results regarding shift workers’ eating habits in the current literature. They may also help to improve preventive interventions for shift workers by tailoring advice to the schedule type.

### 4.1. Heterogeneity of Work Schedules and Impact on Dietary Intakes

When all types of night schedules were combined in the analysis, participants in our study had similar total energy intakes on day and night shifts. This result is in line with most previous studies [16,30,31,39,52] but may hide significant differences in total energy intake depending on the schedule type. Our findings show that some types of night schedules lead to increased energy intake and that intakes during recovery days were far from nutritional recommendations. Even if the impact of eating habits on health depends more on average than acute intake [53], our results show that studies on shift workers’ eating habits should differentiate intake with respect to different schedule types and include days following night shifts and days off. Food intake in the day following a night shift was particularly low in key nutrient and food groups. Workers’ sleep debt and tiredness may decrease their motivation to prepare meals and encourage them to turn to ready-to-eat, often less healthy options. Several studies have shown that hunger increases and satiety decreases after a night shift or a shortened night of sleep [54]. Likewise, food preferences, appetence and food choices are modified toward more sweetened and fatty foods [55,56]. A split-night schedule includes more working hours than other night shifts, which could explain the higher energy intake. Similarly, recovery days may include more sleep than other days. We could not take sleep duration into account in our analysis, but it could explain the lower energy intake on that day. However, even with lower overall intakes, food group intake was unbalanced and might contribute to unhealthy eating habits. On top of food intake, different work schedules certainly also impact physical activity and sleep patterns. We did not record these behaviors, but we anticipate that schedules allowing to sleep at least part on the night, such as evening or half-night schedules, would disrupt them less and allow workers to better keep their routine. On the other hand, a split-night schedule imposes a high workload within 24 h and certainly have the worse impact on sleep, fatigue and physical activity. Such sub-optimal lifestyle behaviors, directly related to the constraints of their schedules, increase the risk of chronic diseases of shift workers, if continued in the long term [25].

### 4.2. Heterogeneity of Profession Types

Shift work is current in types of services, such as first response, security, transportation, care or industry. Besides organizational differences, shift workers also have different professions and belong to different cultures. However, the professional culture may significantly impact eating habits. For example, Dobson et al. (2013) showed that firefighters appear to have created an eating culture in the fire station that includes cooking large amounts of cheap and filling food to withstand frequent call interruptions or snacking on high caloric foods to remain alert around the clock [57]. In our sample, the profession did not significantly impact the results, but most of our sample belonged to the culture of first responders. Results may therefore differ in other professions.

### 4.3. Adjustment of Total Energy Intake

Variations in absolute intake of nutrients in individuals may reflect differences in the composition of food intake or, for the same composition, different levels of total energy intake. The level of energy intake may be a primary determinant or a confounder of a disease; therefore, it is usual to adjust for total energy intake in statistical analysis to ensure that the outcome is not due to the calories consumed [53]. However, for some food groups or nutrients, an absolute intake is warranted, and this recommended intake does not vary according to total energy intake. For example, in adults, fruit and vegetable intake should reach 600 g per day [44], and fiber intake should reach 25 to 30 g per day [58]. Additionally, alcohol intake should not exceed 20 g per day for men regardless of total energy intake [59]. For other nutrients, the relative proportion of intake compared to other nutrients is key. For example, saturated fatty acids should not exceed 10% [60], or added sugar 5%–10% [61], of total energy intake. Therefore, a thorough analysis should focus on absolute intake and adjust for total energy intake. In our sample, participants working split nights had healthier intakes of micronutrients and fiber. However, this finding occurred due to a high total energy intake associated with an overall unbalanced intake, illustrated by a high saturated fatty acid or sodium intake and a lower healthy eating score. On the other hand, on days following night shifts, workers had an overall better diet composition but with a clear insufficiency in several nutrients such as fiber, magnesium and vitamin C. Both situations lead to unhealthy food intake and could represent an additional risk factor for chronic disease development. Regardless of total energy intake, meal frequency may also play an important role [62]. Shift workers tend to eat more frequently [30]. However, in the context of a hypercaloric diet, an increased meal frequency is associated with metabolic disturbances [63].

### 4.4. Preventive Interventions for Shift Workers

Health-related interventions among shift workers have traditionally targeted four areas: (1) controlled light and dark exposure, (2) shift schedule changes, (3) behavioral and lifestyle interventions and (4) pharmacological aids to promote sleep or alertness [64]. A recent systematic review revealed only seven interventions promoting healthier food and/or physical activity habits among employees working “around the clock” hours [65], showing small to moderate effect sizes in several measures. This finding is supported by a systematic review of worksite interventions focused on employees’ diets, which also showed that these programs are associated with moderate improvements in dietary intake [66]. An increasing number of experts are calling for the development and evaluation of well-designed health promotion programs, including the promotion of healthy eating among shift workers [67,68]. A challenge remains due to the lack of consensus on specific nutritional recommendations for shift workers. A large body of evidence shows that metabolism of carbohydrates and lipids is disturbed during the night [69,70], supporting the argument that night workers should avoid or limit eating during the night [31]. Gupta et al. (2017) studied the effects of timing of eating on simulated driving performance across four simulated night shifts and concluded that for optimal performance, shift workers should consider restricting food intake during the night [71]. Our data showed that individuals are not consuming many calories during the night, but fasting at night may be unrealistic for some shift workers, especially those with high physical activity. Prevention efforts should therefore be designed to include the development of strategies to minimize metabolic disturbances due to food intake at night.

Moreover, if circadian rhythms impact important digestive and metabolic processes, conversely, eating at regular times is one of the factors implicated in the resynchronization of internal peripheral clocks [22,72]. Evidence suggests regular meals play the role of “Zeitgebers” [70]. Indeed, nutrients and hormones secreted for digestion act as metabolic signals for peripheral tissues. Some preliminary animal studies also show that the type of diet (high or low in fat) may also influence peripheral clocks [66]. Yet, in our sample, shift workers’ eating habits tended to follow nutritional recommendations less closely compared to the general population. Our participants working one, two or three or more nights had, for example, a lower consumption of fruit, vegetables and dairy, as well as a higher consumption of meat, fish and eggs than the Swiss general population [73]. The mean healthy eating score (PNNS-GS score) of our 3 groups (respectively 5.95 ± 1.39, 6.46 ± 1.52, and 6.75 ± 1.67) was also lower than the results of a large French study that found a median score of 7.55, and the lowest quartile at or below 6.25 [45] in a general population. Therefore, while waiting for more specific and tailored advice for shift workers, classical healthy eating promotion may improve eating habits and structure, help limit circadian disruption and ultimately contribute to a reduced disease burden in this population. Our results suggest in particular promoting consumption of nutrient-dense food such as fruits, vegetables, whole grains and plant-based protein sources. In addition, special consideration should be given to help shift workers plan and structure their food intake and choose healthy and convenient food before, during and after night shifts. In addition, preventive measures should target shift workers regardless of the number of nights worked, as even one night shift is associated with unbalanced food intake. 

The food offer for night workers is often poor, with low access to healthy dining options and fresh foods and energy-dense snack foods easily available through vending machines [74]. Therefore, environment-level interventions should complement intrapersonal approaches to support healthy choices [75]. Future preventive intervention could, for example, focus on improving healthy food availability or offering a dedicated place and time for workers to eat their meals.

### 4.5. Study Strengths and Limitations

The strength of this study is its reliance on the analysis of a sequence of consecutive shifts and days off, which resulted in more than 365 24-h food records and allowed a comparison of shift workers’ eating habits according to the number of nights worked and schedule type. Participants were strongly involved and motivated, resulting in high-quality data and few missing data. This motivation is especially important for the 5-day food record, which otherwise represents a weak spot in data collection.

This study also has several limitations. Body fat mass was estimated with impedance and was not based on a DXA or CT scan measurement. In this study, we did not measure circadian rhythm. Similarly, we did not collect information on sleep pattern, which could influence food intake. Our design did not include fixed day workers. Therefore, we can only compare mean dietary intakes among workers with more or less irregular schedules, and we cannot isolate the effect of shift work compared to day work. Our sample included only males, and participants of our study worked fast, clockwise-rotating shifts. Therefore, the external validity of our results may be limited compared, for example, to other common shift systems in industry or a hospital with shifts alternating between weeks of mornings, evenings and nights. Moreover, our finding relating to split nights was based only on a subsample of our data, police officers, who worked this type of schedule. This finding does not rely on a comparison of this study group to the others but relies rather on a mixed model combining data from all participants and schedule types. Therefore, we cannot exclude that the same analysis in other workers may have brought different results. We observed that the mean dietary intakes of shift workers with one work night tended to be less healthy than those working three nights or more. This non-significant result may occur due to a lack of power and should be retested in a larger sample.

## 5. Conclusions

Eating habits are an important determinant of metabolic and chronic disease development in shift workers. Our results show that even one night during the shift period is associated with unhealthy food intake. Moreover, the quantity, distribution and quality of food intake differ between shift schedules. Therefore, prevention interventions need to include tailored preventive messages based on schedule types and help shift workers plan and anticipate their food intake before, during and after night shifts, regardless of the number of nights worked. Future studies should also investigate adherence to such interventions, especially barriers and facilitators to behavior change strategies.

## Figures and Tables

**Table 1:** Participants’ characteristics (mean ± SD) by worksite and for the total sample.

	Airport Employees (*n* = 8)	Professional Firefighters (*n* = 28)	Police Officers (*n* = 29)	Total Sample (*n*= 65)
Age (years)	39.6 (8.2)	40.2 (6.3)	36.7 (7.0)	38.6 (7.0)
Years of service (years)	10.2 (7.1)	17.0 (6.3)	11.3 (7.9)	13.7 (7.6)
Body Mass Index (kg/m^2^)	27.4 (2.9)	27.1 (3.7)	26.7 (2.9)	26.9 (3.3)
Body fat mass (%)	26.8 (6.7)	23.8 (6.5)	23.0 (6.8)	23.8 (6.7)
Following a diet (n participants)	0	5	6	11 (17%)
Duration of the study period (days)	5.2 (0.6)	5.0 (0.6)	6.4 (0.5)	5.7 (0.9)
1 night worked (n participants)	3	0	15	18 (17.7%)
2 nights worked (n participants)	0	28	8	36 (55.4%)
3 nights worked or more (n participants)	5	0	6	11 (16.9%)

**Table 2:** Daily mean intakes (SD) of energy, nutrients and food groups; mean healthy score (PNNS-GS) and eating behaviors scores of shift workers working one, two, or three or more nights during the study period (5 or 6 consecutive days).

	1 Night (*n* = 18)	2 Nights (*n* = 36)	3 Nights or More (*n* = 10)	*p*
Energy (kcal)	2221 (602)	2311 (621)	2423 (581)	0.54
Energy per kg (kcal)	25.9 (5.9)	27.7 (8.4)	30.0 (8.4)	0.38
Proteins (% of TEI)	19.4 (4.2)	18.3 (4.7)	18.7 (5.8)	0.68
Lipids (% of TEI)	35.8 (3.8)	35.6 (6.2)	33.5 (4.6)	0.44
SFA (% of TEI)	13.7 (1.6)	13.9 (3.1)	12.2 (3.4)	0.42
CHO (% of TEI)	43.2 (5.6)	44.8 (8.3)	45.8 (6.6)	0.39
Fiber (g)	17.9 (4.7)	19.0 (7.5)	23.8 (11.5)	0.17
Vitamin C (mg)	85.2 (30.2)	106.5 (67.2)	102.3 (109.4)	0.42
Calcium (mg)	725 (254)	804 (353)	786 (305)	0.53
Magnesium (mg)	236 (73)	262 (125)	303 (161)	0.22
Fruits (serv)	0.63 (0.42)	0.80 (0.79)	1.10 (1.05)	0.45
Vegetables (serv)	1.54 (0.76)	1.76 (1.04)	1.48 (0.98)	0.69
Starchy foods and grains (serv)	3.06 (1.20)	3.31 (1.23)	3.57 (1.0)	0.51
Whole grains (serv)	0.31 (0.34)	0.24 (0.41)	0.71 (0.86)	0.10
Meat, fish, eggs (serv)	2.60 (1.61)	2.28 (1.43)	2.42 (1.19)	0.84
Dairies (serv)	1.33 (0.83)	1.41 (0.96)	1.18 (0.54)	0.59
Added fats (serv)	2.48 (1.29)	3.24 (2.23)	2.87 (3.89)	0.47
Added sugar (g)	45.0 (28.9)	49.2 (33.3)	39.5 (23.9)	0.71
Hydration (water, tea, coffee) (mL)	1466 (679)	1470 (626)	1674 (895)	0.84
Sugar-sweetened beverages (mL)	178 (201)	231 (385)	179 (206)	0.99
Alcoholic beverages (serv)	1.75 (1.96)	1.43 (2.57)	0.93 (1.33)	0.45
PNNS-GS score	5.95 (1.39)	6.46 (1.52)	6.75 (1.67)	0.35
Meals (frequency)	5.96 (1.78)	6.34 (2.51)	8.21 (2.79)	0.11
Food and beverages (other than water)	13.2 (2.5)	13.2 (3.8)	13.9 (4.0)	0.70
DEBQ Restrained eating	2.53 (0.45)	2.55 (0.83)	2.89 (0.65)	0.47
DEBQ Emotional eating	1.68 (1.17)	1.70 (0.92)	1.37 (0.49)	0.66
DEBQ External eating	2.96 (0.73)	2.91 (0.44)	2.71 (0.61)	0.43
IES Intuitive eating scale	3.46 (0.55)	3.58 (0.60)	3.68 (0.50)	0.74
IES Eating for physical rather than emotional reasons	3.77 (0.98)	3.94 (0.82)	4.18 (0.80)	0.63
IES Reliance on hunger and satiety cues	3.20 (0.92)	3.24 (0.77)	3.33 (0.98)	0.60
IES Unconditional permission to eat	3.25 (0.78)	3.39 (0.91)	3.18 (1.16)	0.81

TEI = total energy intake, SFA = saturated fatty acids, CHO = carbohydrates, serv = serving, DEBQ = Dutch Eating Behavior Questionnaire, IES = Intuitive Eating Score.

**Table 3:** Mean intakes (SD) of energy, nutrients and food groups of shift workers during different schedule types.

	Day Shift (*n* = 117)	Evening Shift (*n* = 16)	Half Night Shift ^1^ (*n* = 18)	Split Night Shift ^2^ (*n* = 19)	Night (*n* = 69)	Day Following Night (Recovery) (*n* = 53)	Day Off (*n* = 73)	*p*
Energy (kcal)	2398 (816) ^B,C^	2107 (631) ^B^	2277 (565) ^B^	3370 (1764) ^A^	2233 (883) ^B^	1993 (727) ^B,D^	2379 (866) ^B^	<0.0001
Energy per kg (kcal)	28.6 (11.0) ^B^	24.0 (7.4) ^B^	28.9 (7.5) ^B^	40.0 (19.7) ^A^	26.6 (11.2) ^B^	23.7 (9.0) ^B^	28.3 (10.0) ^B^	<0.0001
Proteins (% of TEI)	18.5 (5.9)	19.7 (8.3)	20.8 (8.9)	19.8 (6.3)	18.0 (6.8)	20.8 (9.4)	19.3 (7.3)	0.16
Lipids (% of TEI)	34.9 (8.8)	32.4 (8.7)	34.4 (6.2)	34.4 (7.7)	33.7 (9.5)	34.5 (11.5)	37.4 (7.6)	0.15
SFA (% of TEI)	13.2 (4.1)	12.0 (3.8)	12.3 (5.7)	14.2 (4.9)	13.9 (5.7)	12.4 (5.8)	14.1 (4.5)	0.26
CHO (% of TEI)	44.9 (10.5)	46.2 (14.4)	42.8 (9.1)	44.2 (6.7)	46.6 (10.8)	45.0 (10.8)	41.6 (10.9)	0.50
Fiber (g)	20.5 (10.2) ^B^	18.2 (6.0)	21.7 (9.3)	27.4 (18.1) ^B^	17.8 (9.5)	15.5 (7.5) ^A^	21.2 (15.0)	0.0002
Vitamin C (mg)	120 (116)	74 (90)	103 (117)	126 (116)	95 (88)	88 (89)	86 (68)	0.001
Sodium (mg)	3588 (2753)	2561 (821)	3456 (1513)	4794 (2359) ^A^	3001 (1711)	2531 (3184) ^B^	2837 (1795) ^B^	<0.0001
Calcium (mg)	813 (399) ^B^	739 (622)	595 (336) ^B^	1354 (1025) ^A^	748 (455) ^B^	633 (402) ^B^	821 (714) ^B^	0.0001
Magnesium (mg)	280 (133)	247 (161)	266 (148)	391 (310) ^A^	241 (130) ^B^	231 (127) ^B^	270 (208) ^B^	0.0003
Alcohol (g)	12.4 (23.9) ^D^	4.8 (9.1)	2.9 (7.1)	8.7 (12.6)	5.0 (12.7) ^A^	30.4 (57.3) ^B,C^	19.3 (33.6) ^B^	<0.0001
Fruits (serv)	0.98 (1.31)	0.93 (1.08)	0.81 (0.99)	0.71 (0.80)	0.86 (1.18)	0.50 (0.81)	0.77 (0.99)	0.04
Vegetables (serv)	1.87 (1.50)	1.31 (1.73)	1.83 (1.80)	1.70 (1.03)	1.64 (1.35)	1.49 (1.21)	1.44 (1.33)	0.21
Fruits and vegetables (serv)	2.85 (2.07) ^B^	2.23 (1.79)	2.64 (2.24)	2.41 (1.42)	2.49 (1.90)	1.99 (1.42) ^A^	2.21 (1.75)	0.001
Starchy foods and grains (serv)	3.44 (2.30) ^B^	3.29 (1.92)	3.21 (1.81)	5.33 (3.27) ^A^	3.40 (2.01)	2.64 (1.88) ^B^	3.08 (1.95) ^B^	0.0004
Whole grains (serv)	0.31 (0.60)	0.31 (0.38)	0.39 (0.56)	0.59 (0.85)	0.30 (0.76)	0.21 (0.47)	0.49 (0.89)	0.21
Meat, fish, eggs (serv)	2.33 (1.70) ^B^	2.87 (2.88)	3.27 (1.96)	5.10 (7.47) ^A^	2.03 (1.24) ^B^	2.19 (1.56) ^B^	2.39 (1.94) ^B^	<0.0001
Dairies (serv)	1.37 (1.27)	1.33 (1.13)	0.62 (0.70)	2.51 (2.86) ^A^	1.30 (1.33)	1.03 (1.21) ^B^	1.54 (1.76)	0.002
Hydration (water, tea, coffee, diet soda) (mL)	1598 (961) ^D^	1184 (901)	1687 (1074)	2166 (994) ^A^	1458 (859) ^B^	1264 (735) ^B,C^	1491 (899) ^B^	<0.0001
Sugar-sweetened beverages (mL)	246 (454)	196 (247)	79 (148)	195 (292)	301 (484)	168 (381)	122 (196)	0.04
Number of eating occasions	7.0 (3.3) ^C^	6.5 (2.0)	8.2 (3.8) ^E^	8.6 (3.8) ^A,D^	6.6 (3.0) ^B,D^	5.5 (2.7) ^B,D,F^	5.7 (2.5) ^B,D,F^	<0.0001

^1^ shift ending at 0 a.m. or 1 a.m., ^2^ shift from 6 a.m. to 12 p.m. and 7 p.m. to 6 a.m. TEI = total energy intake, SFA = saturated fatty acids, CHO = carbohydrates, serv = serving. ^A,B^ Significant differences between groups (Post-Hoc analyses) where results labelled A are significantly different from B (same for C, D and E, F).

## Supplementary Materials

The following are available online at https://www.mdpi.com/2072-6643/12/4/919/s1, Table S1: Proportion (%) of energy intake for each 8-hour period during different schedule types.
