# Supplementary material for: Energy, Nutrient and Food Intakes of Male Shift Workers Vary According to the Schedule Type but Not the Number of Nights Worked
Source: Nutrients. 2020 Mar 27;12(4):919. doi: 10.3390/nu12040919 (PMC7230522; doi:10.3390/nu12040919)
Supplement: Supplementary file 1 [file nutrients-12-00919-s001.pdf]

nutrients

Supplementary Material

Energy, Nutrient and Food Intakes of Male Shift Workers Vary According to the Schedule Type but Not the Number of Nights Worked

Sophie Bucher Della Torre [1,*], Pascal Wild [2,3], Victor Dorribo [2], Brigitta Danuser [2,†] and Francesca Amati [4,†]

Table S1. Proportion (%) of energy intake for each 8-hour period during different schedule types.

	Day	Evening	Half Night	Split night	Night	Recovery	Off
5am–1pm	48	34	33	37	33	29	36
1pm–9pm	44	52	43	26	53	53	56
9pm–5am	8	13	24	37	14	18	8
